# Mobilising cultural heritage for locally owned adaptation

**DOI:** 10.1038/s44168-025-00309-3

**Published:** 2025-11-19

**Authors:** Kate Donovan, Rowan Jackson, Siona O’Connell, Dulma Karunarathna, Arry Retnowati, Esti Anantasari, YoungHwa Cha, Dominique Niemand, David C. Harvey, Andrew Dugmore

**Affiliations:** 1 Edinburgh Climate Change Institute, School of GeoSciences, University of Edinburgh, Edinburgh, Scotland; 2 Global Academy of Agriculture and Food Systems. University of Edinburgh, Edinburgh, Scotland; 3 Faculty of Humanities, University of Pretoria, Pretoria, South Africa; 4 Centre for Asia Pacific Initiatives, University of Victoria, Victoria, VIC Canada; 5 Centre for Asia Pacific Studies, Universitas Gadjah Mada, Yogyakarta, Indonesia; 6 Department of Civil and Environmental Engineering, University of Strathclyde, Glasgow, Scotland; 7 Department of Archaeology and Heritage Studies, Aarhus University, Aarhus, Denmark; 8 School of GeoSciences, University of Edinburgh, Edinburgh, Scotland

**Keywords:** Cultural and media studies, Cultural and media studies, Development studies, Environmental studies, Geography, Geography, History, History, Sociology

## Abstract

Climate change adaptation planning and implementation has been criticised for following linear steps that can limit local suitability, scalability and sustainability. We argue that meaningful climate change adaptations incorporate a diversity of voices using cultural heritage for situated and multi-generational interventions. Here, we present examples of risk narratives and adaptive strategies developed through engagement with cultural heritage, balancing knowledge of environment with local livelihoods, histories, values and meaning.

## Challenging adaptation

Few societies have implemented adaptation plans at the pace or scale required to address exposure of climate change impacts^1^. Conventional approaches to risk communication and ‘community-based’ adaptation frequently fail to gain traction^2^ due to limited funding, capacity building or scalability^3–5^. Concepts of community, place or the local are commonly used across disaster management and climate change adaptation (CCA) yet, despite their appeal, they are problematic and often vaguely deployed^6^. And, despite the discourse relating to local scale adaptation^7^, few studies have demonstrated significant impact that operates within the social and cultural fabric of day-to-day life^4,8,9^.

CCA is a complex transdisciplinary challenge requiring contributions from hazard modelling through to behavioural sciences^10^. We argue that while contributions to global change and adaptation research have increased, adaptations remain either too general in application or ill-applied to cultural values at the local scale. Furthermore, drawing on post-humanist approaches within Critical Heritage Studies, we highlight the need to make room for how everyday lifeworlds are situated within the context of an entanglement of human and non-human agencies that scaffold the experience of ‘changefulness’ over time^11,12^. Local expressions of climate impacts and adaptation are often missing from CCA literatures that tend to describe the implementation of adaptation as: (1) fixed in time, (2) operationalized and leveraged in a mechanistic way (upon a ‘community’ rather than within a complex and shifting set of social arrangements), and (3) consider culture and people as static assets rather than as dynamic and relational beliefs, values, practices and histories that comprise values and meanings. The enactment of adaptation needs to reflect the dynamic, relational properties of people and where they live, capturing historical and cultural histories of place and on-going change^13^.

## The role of heritage

Place-sensitive and heritage-informed approaches have significant potential to underpin and sustain adaptation and promote climate-responsive learning^14–16^. This, we argue, is because cultural heritage is not simply a tangible asset to be protected but a critical tool comprising of traditional and indigenous knowledge, belief systems, and practices that enhance understandings of climate risk and inform the shaping of adaptation options, particularly at a local scale.

In a recent Nature Climate commentary, Simpson^17^ called for a decolonization of climate change-heritage research to recognise the diversity of cultural expressions impacted by, and offering adaptive capacities in response to, global change. Cultural heritage can inform successful adaptation because it embodies existing forms of knowledge, ongoing adaptive capacities and local understandings of ‘nature’, the organisation of resource systems, and sociocultural arrangements that deliver legitimacy and collective support^18^. The intangible assemblage of capacities heritage offers by contextualising how value and risk associations are perceived by local actors and, in turn, what adaptations should be planned has significant potential, but a material bias towards monuments, archaeological sites and buildings predominates across heritage literature and characterise heritage as passive and ‘at-risk’^19^. Despite advances in capturing heritage value and significance via for example The Burra Charter^20^, few risk assessment approaches consider intangible heritage and most studies to date focus on the Minority Geographies of North America, Western Europe, and Australia^16^. Yet every person has a distinct cultural heritage that provides context to our motivations, our sense of place and history, and our attitude to the future^21^. To therefore understand locality for climate change action, requires practitioners and researchers to explore the messy reality of life, breaking the frameworks that have provided neat solutions. There is an opportunity to rethink western constructs of climate risk assessment and explore a diversity of voices across the heritage-risk-adaptation nexus for improved sustainable development and resilience.

We respond directly to Simpson’s^17^ call for new methods to integrate heritage into climate assessments, avoiding an overt ‘Eurocentric view’ of climate change-heritage research through multi-layered and non-hierarchical dialogue led by local communities and research institutes in South Africa, Sri Lanka and Indonesia.

## Methods

### Heritage as a tool for action

In Sri Lanka, interviews and workshops in rural settlements captured narratives of indigenous environmental knowledge systems (IKS). These narratives highlighted interconnected threads of heritage colonization, gender and rural livelihoods. Research in rural villages identified marginalised environmental knowledge held specifically by women, which was underappreciated in the context of socio-economic pressures to modernise. This finding led to the co-creation of a series of engagements with groups of women to better understand and share the skills, knowledge and values of their ancestors concerning environmental management and disaster preparedness. They identified specific knowledge relating to the Mee Tree (*Madhuca longifolia*) and its multiple resources^22^. This tree is valued for its diverse uses, including traditional medicine and oil extraction. Based on the knowledge of elders, hundreds of guides were produced for school aged children so that they could cultivate saplings and benefit from the trees adaptative capacities.

In Yogyakarta, Indonesia, interviews and focus group discussions were conducted to better understand and identify the heritage of the city and its natural landscape of river catchments stretching from the Merapi volcanic area southward to the Indian Ocean. People living along the Kali Code (River Code) identified this landscape as their heritage asset. The cultural value of an urban river system thus enabled discussions on community engagement. That led to the capturing of narratives around landscape changes, including how external influences such as riverbank construction for tourism-centred developments led to behavioural shifts toward the river, transforming it from cultural and environmental significance to a place to discard rubbish. The study sought to understand the correlation between climate-related hazards and the cultural heritage which connects people to their environment. We found that people wanted to share their traditional practices of ‘working together’ across generations to find a way of improving awareness and reducing the polluting behaviors. This resulted in a youth-led theatre production and comic book to share knowledge with others and communicate the need to care for the river as a critical heritage asset.

In South Africa, workshops, a household survey and a series of interviews were conducted in Elandskloof in the Cederberg, which forms part of the Cape Floral Kingdom World Heritage Site. Significant as the site of the first ’successful’ land restitution case in democratic South Africa^23^, Elandskloof is now an impoverished settlement where IKS loss has disconnected the land from daily life and fractured the community. Through the lens of food heritage, new insights were gleaned about how heritage practices may pave the way to intergenerational knowledge exchange, the construction of a healthier community, and a pathway toward sustainable development. Here the heritage-risk narrative highlights the violent and forced removals from the land that have led to significant vulnerabilities for communities across South Africa. By placing heritage at the centre of discussions with the people of Elandskloof they identified ways to address their vulnerabilities through sharing and growing new local knowledge on horticulture, developing their own plant nursery and sharing the responsibility for its management between generations. They successfully implemented this work with little external support and continue to maintain their heritage food garden, which in their words has become a ‘sign of hope for the future’.

### Lessons for locally led adaptation

**Fig. 1:**
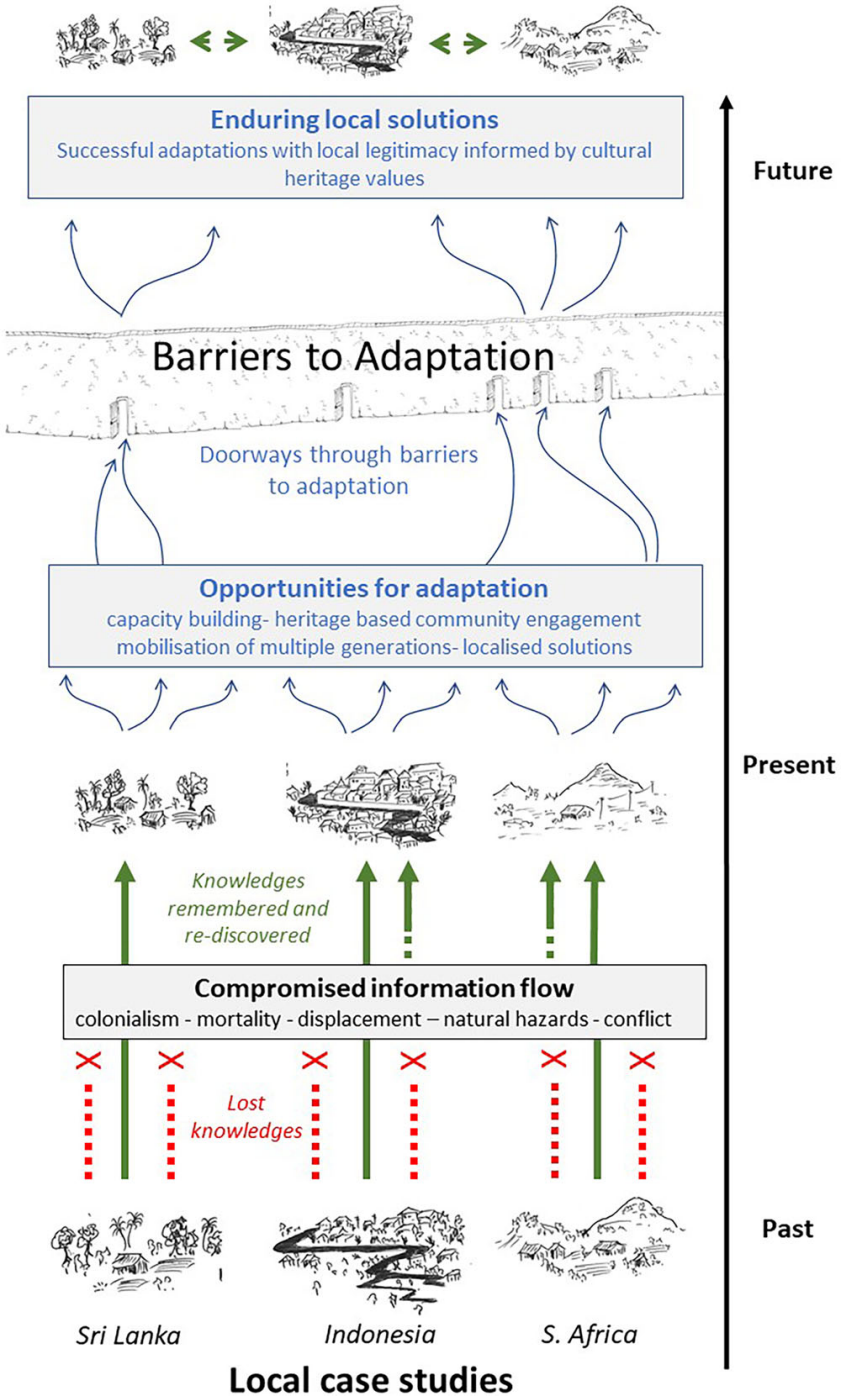
Illustrating the challenges facing people living in highly vulnerable settings and opportunities of heritage as a tool for adaptation.

These three distinct studies have common opportunities for adaptation and wider disaster management. By refocussing adaptation efforts on cultural heritage value, significant progress was made in identifying their perceptions of the climate risk-scape, as well as important actions that were viable, situationally appropriate and, essentially, locally owned (Fig. 1).

The first lesson was recognising how place-based or local knowledge can be lost and found after displacement or in situ oppression. In all three locations, knowledge and skills had been displaced through the adoption of ‘western’ ideals. By focussing on cultural heritage value, people were able to connect and mobilise their beliefs and practices for actions that are locally owned, culturally acceptable and sustainable. For example, in Sri Lanka, a self-organising group of women shared their traditional Mee Tree oil extraction skills and knowledge of its multiple applications from medicine to fuel.

Secondly, sharing of this knowledge and the demonstration of its application led to collective ownership of both the challenge and multiple opportunities. This co-creation began a healing process in Elandskloof that demonstrated to external bodies, such as the local government, that this community was not a ‘lost cause’ and deserves further support to access basic services. In Indonesia, it has led to a communication campaign involving multiple generations working together to share their environmental knowledge and improve the water quality of the River Code.

Thirdly, for the international research team it drove a deconstruction of conventional research practice towards a cross-disciplinary way of sharing knowledge and action. Refocussing discussions on what people value and consider to be heritage, identified ways to address a triple challenge for contemporary policy makers: i) understanding the benefits of place-based knowledge; ii) mobilising this knowledge and iii) empowering local people to continue the development and refinement of their knowledge. This work evidenced that the complex relationships between people and natural systems are dynamically organized and structured across different scales of space and time, creating a panarchy of interactions mediated by information flow.

Through the critical reframing of climate and disaster vulnerability of heritage towards capacity, the resilience of cherished places and people’s cultural knowledge, views and values are better understood. We therefore join the call for researchers and practitioners to consider cultural entry points for locally owned and enacted adaptations.

## Data Availability

The data that support the findings of this study are not openly available due to ethical reasons of sensitivity and are available from the corresponding author upon reasonable request.
